# Academic engagement mediates the association between curiosity and mathematics achievement among Chinese eighth grade students

**DOI:** 10.3389/fpsyg.2026.1906668

**Published:** 2026-09-11

**Authors:** Wen Du

**Affiliations:** School of Mathematics and Statistics, Qinghai Normal University, Xining, Qinghai, China

**Keywords:** academic engagement, deprivation-type curiosity, interest-type curiosity, mathematics achievement, mediation model

## Abstract

**Introduction:**

This study examined how interest-type and deprivation-type curiosity were associated with mathematics achievement through cognitive, behavioral, emotional, and social engagement.

**Methods:**

Drawing on self-determination theory and a multidimensional perspective on academic engagement, we analyzed cross-sectional data from 677 eighth-grade students across six junior secondary schools in Qinghai Province, China. Curiosity and academic engagement were assessed using mathematics-specific self-report measures, whereas achievement was obtained from a unified end-of-term mathematics examination. Structural equation modeling with robust maximum likelihood estimation was conducted while controlling for age, gender, home book resources, and maximum parental educational attainment.

**Results:**

The model showed an acceptable fit (CFI = 0.928, TLI = 0.922, RMSEA = 0.047, SRMR = 0.040). Both curiosity types were positively associated with all four engagement dimensions. Behavioral engagement showed the strongest association with mathematics achievement (β = 0.326), followed by emotional engagement (β = 0.147), and both dimensions accounted for significant indirect associations between curiosity and achievement. Interest-type curiosity also retained a significant direct association with achievement (β = 0.113), whereas deprivation-type curiosity was associated with achievement primarily through engagement-related pathways. Cognitive engagement did not show a significant unique association with achievement.

**Discussion:**

These findings indicate that the two curiosity types are not functionally equivalent and that academic engagement does not operate as a uniform pathway. By identifying behavioral persistence and emotional involvement as the most salient pathways, this study refines the motivation-engagement-achievement framework in mathematics learning.

## 1 Introduction

Curiosity is typically defined as a psychological tendency to actively seek understanding and information when individuals encounter novel, uncertain, or complex situations (Kashdan et al., 2018). A substantial body of research has consistently documented positive associations between curiosity, learning motivation, academic engagement, and academic achievement (Engel, 2011; Jirout et al., 2018; Jirout and Klahr, 2012; von Stumm et al., 2011). However, most of these studies have focused on general cognitive curiosity, with comparatively limited attention to its mechanisms within specific subject domains. Emerging evidence suggests that domain-specific curiosity may play a more direct and critical role in the learning process (Azizmohammadi et al., 2023; Singh and Manjaly, 2022), yet empirical findings in this area remain fragmented.

In the context of mathematics learning, mathematical curiosity is generally regarded as an intrinsic driving force that motivates students to engage in sustained exploration of mathematical concepts and problems. It can be further differentiated into interest-type curiosity and deprivation-type curiosity (Litman, 2005, 2008; Litman and Spielberger, 2003). The former reflects a spontaneous desire to explore mathematical knowledge, whereas the latter is characterized by sensitivity to cognitive uncertainty or knowledge gaps and a motivation to resolve them. Although existing studies have provided preliminary evidence for the positive role of mathematical curiosity in learning performance (Mahama et al., 2023; Shah et al., 2018), the differential mechanisms through which these two types of curiosity influence academic achievement remain insufficiently examined.

Self-determination theory provides a useful foundation for explaining why curiosity may be associated with mathematics achievement through students' engagement in learning (Ryan and Deci, 2000, 2020). From this perspective, intrinsically oriented motivation supports sustained participation because learners experience an activity as interesting, meaningful, or personally involving. However, motivation does not automatically translate into academic performance. Its educational significance depends partly on how it is expressed through students' cognitive, behavioral, emotional, and social involvement in learning.

Although previous studies have linked curiosity to academic engagement and achievement, three issues remain insufficiently addressed. First, curiosity has often been treated as a general motivational tendency, whereas interest-type curiosity and deprivation-type curiosity may reflect different motivational qualities. Interest-type curiosity is characterized by enjoyment in exploring new information, while deprivation-type curiosity is driven by the perceived need to reduce uncertainty or close knowledge gaps. Distinguishing these two forms is theoretically meaningful because they may be associated with different forms of engagement in mathematics learning. Interest-type curiosity may be more closely related to positive emotional involvement and voluntary participation, whereas deprivation-type curiosity may depend more strongly on students' ability to regulate uncertainty and sustain effort during problem solving.

Second, existing studies have rarely examined whether different dimensions of academic engagement play distinct roles in linking curiosity with achievement. Academic engagement is multidimensional, encompassing cognitive, behavioral, emotional, and social engagement. These dimensions capture related but distinguishable forms of involvement in mathematics learning, including strategic processing, persistence and participation, affective involvement, and peer interaction. Examining them separately may therefore provide a more precise account of the engagement pathways through which curiosity is associated with mathematics achievement.

Third, educational context may shape the associations among curiosity, engagement, and achievement. Chinese junior secondary students learn mathematics within a highly structured and assessment-oriented system, while schools in western regions such as Qinghai Province remain underrepresented in educational psychology research. Examining the proposed relationships in this context can therefore extend existing evidence beyond predominantly Western and more economically developed settings.

Accordingly, the present study examined the associations among interest-type curiosity, deprivation-type curiosity, cognitive, behavioral, emotional, and social engagement, and mathematics achievement among Chinese eighth-grade students. Drawing on self-determination theory and a multidimensional engagement perspective, the study tested whether the four engagement dimensions accounted for distinct indirect associations between the two forms of curiosity and mathematics achievement. The study makes three contributions. First, it differentiates the academic roles of interest-type and deprivation-type curiosity rather than treating curiosity as a unitary motivational tendency. Second, it examines the four engagement dimensions simultaneously, allowing them to be evaluated as distinct rather than interchangeable pathways. Third, it extends the motivation–engagement–achievement framework to an underrepresented western Chinese mathematics context using achievement data from a unified end-of-term examination.

## 2 Literature review

### 2.1 The association between curiosity and mathematics achievement

Mathematics fundamentally involves the identification, application, and extension of patterns, rules, and axioms, requiring logical reasoning and symbolic representation to understand both tangible and abstract properties of numbers, shapes, and spatial relationships (Bôcher, 1904; Carlson et al., 2006). These cognitive processes are often stimulated by curiosity, as students develop an intrinsic drive to ask “why” and “how,” prompting them to construct models, explore multiple solution strategies, and generate their own mathematical questions rather than relying on rote procedures or repetitive calculations (Boaler and Selling, 2017; De Villiers, 2003).

Empirical research further suggests that curiosity is not only a starting point for mathematical learning but also a reliable predictor of academic achievement. For instance, early longitudinal studies have shown that children's curiosity levels are significantly associated with their kindergarten mathematics performance, accounting for approximately 10% of the variance (Shah et al., 2018). Recent analyses of PISA 2022 data have also identified a positive association between intellectual curiosity and science achievement, providing complementary evidence that curiosity is relevant to performance in cognitively demanding academic domains (Khurma et al., 2025).

Muis et al. (2015), in their cognitive–affective model, conceptualized curiosity as a positive epistemic emotion that may support mathematical problem solving by encouraging strategy use and metacognitive monitoring. Their model highlights the dynamic interplay among curiosity, confusion, frustration, enjoyment, and boredom during learning. This perspective is supported by classroom-based research showing that cycles of confusion and resolution can accompany productive mathematical problem solving (Di Leo et al., 2019).

Taken together, these findings suggest that curiosity may support mathematics learning by directing attention toward unresolved problems, sustaining exploration, and promoting the use of cognitive and metacognitive strategies (Di Leo et al., 2019; Muis et al., 2015; von Stumm et al., 2011). Problem-posing pedagogy provides one instructional context in which such processes may be encouraged. By generating mathematically meaningful questions, exploring alternative strategies, and reflecting on unresolved ideas, students can move beyond routine procedural activity toward more exploratory forms of mathematical thinking (Brown and Walter, 2005; Knuth, 2002; Silver, 1994). Experimental evidence also suggests that curiosity-oriented mathematical activities may support students' mathematical creativity (Setiawan and Surahmat, 2022).

### 2.2 Academic engagement as a mediator

Curiosity, as a relatively stable motivational disposition, promotes learning processes through cognitive exploration and information seeking, and is modestly to moderately positively associated with academic achievement (Berlyne, 1960; Litman, 2005; von Stumm et al., 2011). Its influence is often exerted indirectly through mediating mechanisms, particularly by enhancing academic engagement, which in turn contributes to improved achievement (Wu et al., 2018).

In the context of mathematics learning, students' sensitivity to “knowledge gaps” and their drive to resolve them can increase persistence in problem solving, promote deeper processing of solution strategies, and strengthen self-monitoring abilities. These processes foster behavioral engagement (e.g., sustained attention in class, task completion, and active practice) as well as emotional and cognitive engagement (e.g., interest in and perceived value of mathematical tasks) (Fredricks et al., 2004; Pekrun, 2006). Curious learners are more likely to ask questions, generate and test hypotheses, connect current problems to prior knowledge and experiences, and engage in exploratory activities when appropriate (Bowler, 2010; Dewey, 1910; Jirout and Klahr, 2012; Luce and Hsi, 2015).

Recent research has increasingly examined the mechanisms through which curiosity shapes learning processes, particularly its effects on students' emotional states, behavioral engagement, and academic outcomes. Curiosity is widely recognized as a key intrinsic motivational source that drives learning behaviors and academic achievement (Litman, 2005, 2008; von Stumm et al., 2011). Empirical evidence suggests that students with higher levels of curiosity are more likely to adopt deep processing strategies (cognitive engagement), sustain effort (behavioral engagement), experience positive emotions (emotional engagement), and collaborate with peers (social engagement) (Papageorgiou et al., 2025; Zhong et al., 2025). Furthermore, some studies indicate that interest-type and deprivation-type curiosity may differentially activate specific forms of engagement (Whitecross and Smithson, 2023).

As a cognitively demanding and challenging subject, mathematics relies heavily on sustained engagement and effective emotional regulation (Pekrun et al., 2017). However, there is still a lack of systematic empirical evidence on the comprehensive mechanisms through which different types of curiosity influence academic achievement via multiple dimensions of academic engagement.

### 2.3 The present study

The present study developed a conceptual model grounded in self-determination theory and a multidimensional perspective on academic engagement. Interest-type curiosity and deprivation-type curiosity were conceptualized as distinct motivational orientations, while academic engagement was represented by cognitive, behavioral, emotional, and social dimensions. Mathematics achievement was treated as the academic outcome.

The model examined whether the two forms of curiosity showed different patterns of association with the four engagement dimensions and whether these engagement dimensions accounted for distinct indirect associations with mathematics achievement. Interest-type curiosity was expected to be more closely associated with positive emotional involvement and voluntary participation, whereas deprivation-type curiosity was expected to be more strongly related to sustained effort and the resolution of knowledge gaps. By integrating curiosity and multidimensional engagement within a single model, the study aimed to provide a more differentiated account of the motivation–engagement–achievement relationship in mathematics learning.

**Figure 1:**
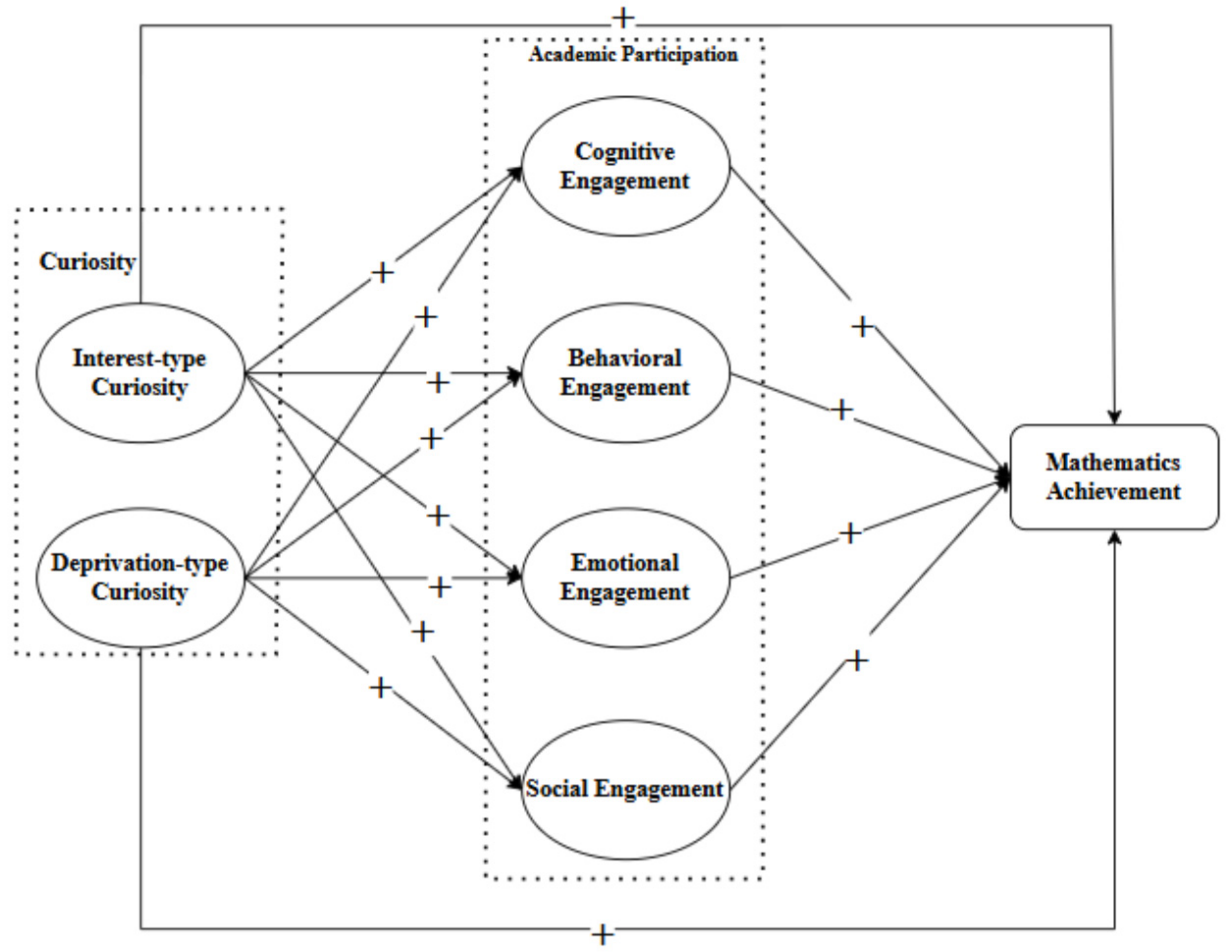
Conceptual model of the relationships among curiosity, engagement, and mathematics achievement.

Based on the theoretical foundations and prior findings in the literature (Azizmohammadi et al., 2023; Leikin et al., 2023; Mahama et al., 2023; Ryan and Deci, 2000; Shah et al., 2018; Wang et al., 2016), this study proposes the following hypotheses (see Figure 1):

**H1a. Interest-type curiosity is positively associated with mathematics achievement**.

**H1b. Deprivation-type curiosity is positively associated with mathematics achievement**.

**H2a. Interest-type curiosity is positively associated with cognitive, behavioral, emotional, and social engagement**.

**H2b. Deprivation-type curiosity is positively associated with cognitive, behavioral, emotional, and social engagement**.

**H3a–H3d. Cognitive, behavioral, emotional, and social engagement are each positively associated with mathematics achievement**.

**H4a–H4d. Cognitive, behavioral, emotional, and social engagement each account for an indirect association between the two curiosity types and mathematics achievement**.

## 3 Research method

### 3.1 Participants and procedure

The analytic sample consisted of 677 eighth-grade students recruited from six junior secondary schools in Qinghai Province, China. The participating schools were purposively selected based on accessibility and their broadly average academic performance within the local education system, followed by intact-class recruitment within the schools. Among the participants, 56.1% were female and 43.9% were male, with a mean age of 14.54 years (SD = 0.83). All participants were enrolled in Grade 8, were native Chinese speakers, and received formal mathematics instruction based on the national curriculum standards. The analytic sample included all students who completed the questionnaire and had a successfully matched end-of-term mathematics examination record.

Data were collected during regular school hours using paper-and-pencil questionnaires administered by trained research assistants. Students completed the curiosity and academic engagement measures independently in quiet, supervised classrooms under standardized instructions, and each administration required approximately 30 min. Participation was voluntary, and students were informed that their responses would remain confidential and would be used solely for research purposes. Research codes rather than names were used to match questionnaire responses with school-provided end-of-term mathematics examination records, after which the analytic dataset was de-identified.

This study was approved by the Research Ethics Committee of the School of Mathematics and Statistics, Qinghai Normal University (approval no. 2025001001) on January 10, 2025. All procedures were conducted in accordance with relevant institutional ethical guidelines and the Declaration of Helsinki and its subsequent amendments. Written informed consent was obtained from both the students and their legal guardians before data collection.

### 3.2 Measures

All self-report measures were administered in mathematics-specific forms, with item wording contextualized to students' experiences of mathematics learning. Where necessary, items were adapted linguistically for Chinese participants and contextually framed around mathematics learning.

#### 3.2.1 Curiosity

Curiosity was assessed using a mathematics-specific adaptation of the 10-item Interest- and Deprivation-type Epistemic Curiosity Scale developed by Litman (2008). The scale comprises two five-item dimensions. Interest-type curiosity reflects the anticipated enjoyment of acquiring new knowledge and exploring unfamiliar ideas, whereas deprivation-type curiosity reflects the desire to resolve uncertainty or close a specific knowledge gap. To align the scale with the domain-specific focus of the present study, the items were contextualized to students' experiences of mathematics learning. For example, an interest-type item was “I enjoy learning about mathematics topics that are unfamiliar to me,” and a deprivation-type item was “When I encounter something in mathematics that I do not understand, I keep thinking about it.” All items were rated on a 4-point scale ranging from 1 (*strongly disagree*) to 4 (*strongly agree*).

The original English-language items were translated into Chinese and reviewed collaboratively by the research team for semantic equivalence, age-appropriate wording, and relevance to mathematics learning. In the present sample, Cronbach's α coefficients were 0.861 for interest-type curiosity and 0.894 for deprivation-type curiosity. All items loaded significantly on their intended latent factors, with standardized factor loadings ranging from 0.688 to 0.764 for interest-type curiosity and from 0.724 to 0.846 for deprivation-type curiosity.

#### 3.2.2 Academic engagement

Academic engagement was assessed using a mathematics-specific adaptation of the Math and Science Engagement Scales developed by Wang et al. (2016), with the multidimensional framework of Fredricks et al. (2004) providing the broader conceptual foundation. The measure comprised four dimensions: cognitive engagement, behavioral engagement, emotional engagement, and social engagement. The measurement model included 8 items for cognitive engagement, 8 items for behavioral engagement, 10 items for emotional engagement, and 7 items for social engagement. All items were rated on a 5-point Likert scale.

Cognitive engagement reflected students' use of strategic and meaning-oriented learning processes in mathematics; behavioral engagement captured effort, persistence, and participation; emotional engagement reflected students' affective involvement in mathematics learning; and social engagement represented participation in peer interaction and collaborative mathematical activity. Example items included “I connect what I learn in mathematics with prior knowledge” for cognitive engagement, “I complete all my mathematics homework on time” for behavioral engagement, “I feel excited when solving mathematics problems” for emotional engagement, and “I enjoy collaborating with classmates in mathematics activities” for social engagement.

The original items were translated into Chinese and reviewed collaboratively by the research team for semantic equivalence, age-appropriate wording, and relevance to mathematics learning. In the present sample, Cronbach's α coefficients were 0.921 for cognitive engagement, 0.927 for behavioral engagement, 0.925 for emotional engagement, and 0.957 for social engagement. All indicators loaded significantly on their intended latent factors. Standardized factor loadings ranged from 0.707 to 0.870 for cognitive engagement, 0.702 to 0.862 for behavioral engagement, 0.726 to 0.900 for emotional engagement, and 0.728 to 0.889 for social engagement.

#### 3.2.3 Mathematics achievement

Mathematics achievement was assessed using students' scores from a unified end-of-term mathematics examination obtained from official school records rather than student self-report. All participating eighth-grade students completed the same examination paper, which was developed in accordance with the national mathematics curriculum standards and administered under comparable school testing conditions. The use of a common examination paper ensured consistency in content coverage and scoring across the six participating schools. Raw examination scores were standardized into z scores before the structural equation modeling analysis, and the standardized scores were used as the observed outcome variable.

### 3.3 Data analysis

Data analyses were conducted using Mplus version 8.3. Descriptive statistics and bivariate correlations were first examined. Because several observed indicators departed from normality, structural equation modeling was estimated using robust maximum likelihood estimation (MLR). Covariance coverage was 1.00 for all variables, indicating that there were no missing values in the analytic dataset.

The model examined the direct and indirect associations of interest-type and deprivation-type curiosity with mathematics achievement through cognitive, behavioral, emotional, and social engagement. Age, gender, home book resources, and maximum parental educational attainment were included as covariates. Indirect associations were estimated using the MODEL INDIRECT command and evaluated using STDYX-standardized estimates, robust standard errors, and 95% confidence intervals. Given the cross-sectional design, these indirect associations were interpreted as statistical pathways rather than evidence of causal mediation.

Model fit was evaluated using χ^2^, the comparative fit index (CFI), Tucker–Lewis index (TLI), root mean square error of approximation (RMSEA) with its 90% confidence interval, and standardized root mean square residual (SRMR). Following established recommendations, CFI and TLI values of approximately 0.90 or higher and RMSEA and SRMR values of approximately 0.08 or lower were regarded as indicating acceptable fit (Hu and Bentler, 1999; Kline, 2023). Because cutoff values should not be treated as rigid rules, model adequacy was evaluated holistically across multiple fit indices and in relation to the theoretical specification of the model (Marsh et al., 2004).

## 4 Results

### 4.1 Preliminary analyses

Table 1 presents the descriptive statistics, internal consistency coefficients, and bivariate correlations among the study variables. Interest-type and deprivation-type curiosity were positively correlated with all four dimensions of academic engagement and mathematics achievement. The four engagement dimensions were also positively correlated with mathematics achievement. These preliminary associations provided support for examining the hypothesized direct and indirect pathways in the structural equation model.

**Table 1:** Descriptive statistics and correlations among variables.

Variable	1	2	3	4	5	6	7
1. Interest-type curiosity	1.000						
2. Deprivation-type curiosity	0.122^**^	1.000					
3. Cognitive engagement	0.370^**^	0.330^**^	1.000				
4. Behavioral engagement	0.401^**^	0.357^**^	0.616^**^	1.000			
5. Emotional engagement	0.422^**^	0.263^**^	0.605^**^	0.618^**^	1.000		
6. Social engagement	0.294^**^	0.237^**^	0.543^**^	0.560^**^	0.610^**^	1.000	
7. Mathematics achievement	0.380^**^	0.266^**^	0.517^**^	0.632^**^	0.577^**^	0.504^**^	1.000
Cronbach's α	0.861	0.894	0.921	0.927	0.925	0.957	
Means	2.380	2.120	3.508	3.479	3.484	3.490	59.222
SD	0.292	0.391	0.734	0.700	0.663	0.552	27.181

Mathematics achievement descriptive statistics are reported in raw-score units; standardized z scores were used in the structural equation modeling analyses. ^**^p < 0.01.

### 4.2 Structural equation model

The covariate-adjusted structural equation model showed an acceptable fit to the data, χ^2^(1032) = 2546.18, *p* < 0.001, CFI = 0.928, TLI = 0.922, RMSEA = 0.047, 90% CI [0.044, 0.049], and SRMR = 0.040. The model explained 23.4% of the variance in cognitive engagement, 26.9% in behavioral engagement, 23.9% in emotional engagement, 13.9% in social engagement, and 39.3% in mathematics achievement.

After controlling for age, gender, home book resources, and maximum parental educational attainment, interest-type curiosity was positively associated with cognitive engagement (β = 0.336, *p* < 0.001), behavioral engagement (β = 0.356, *p* < 0.001), emotional engagement (β = 0.392, *p* < 0.001), and social engagement (β = 0.268, *p* < 0.001). Deprivation-type curiosity was also positively associated with cognitive engagement (β = 0.279, *p* < 0.001), behavioral engagement (β = 0.300, *p* < 0.001), emotional engagement (β = 0.199, *p* < 0.001), and social engagement (β = 0.195, *p* < 0.001).

**Figure 2:**
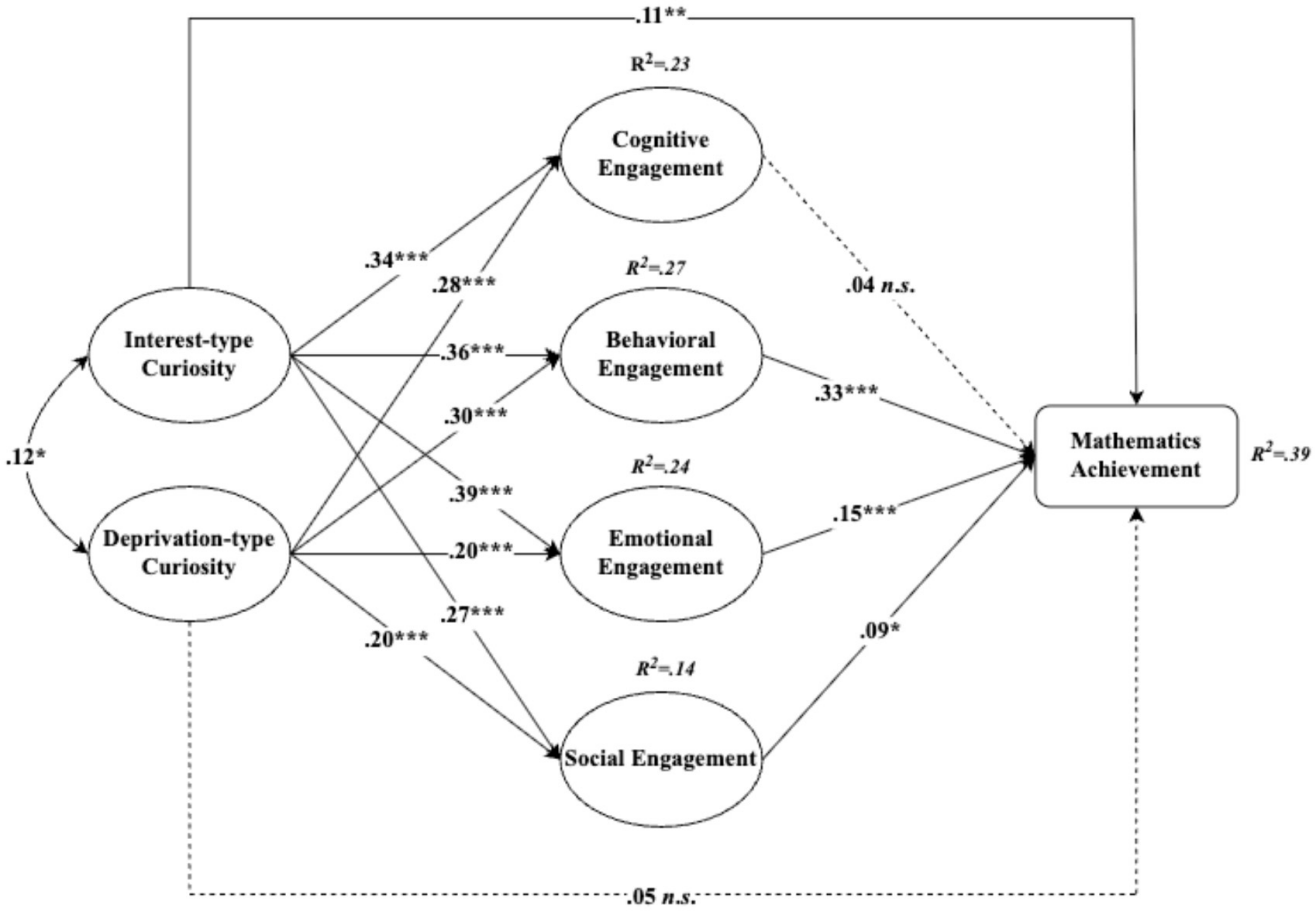
The relationships among curiosity dimensions, academic engagement, and mathematics achievement. *p < 0.05, **p < 0.01, ***p < 0.001.

Behavioral engagement was positively associated with mathematics achievement (β = 0.326, *p* < 0.001), as were emotional engagement (β = 0.147, *p* = 0.006) and social engagement (β = 0.090, *p* = 0.046). Cognitive engagement was not significantly associated with mathematics achievement (β = 0.038, *p* = 0.526). Interest-type curiosity retained a significant direct association with mathematics achievement (β = 0.113, *p* = 0.002), whereas the direct association between deprivation-type curiosity and mathematics achievement was not statistically significant (β = 0.053, *p* = 0.107). The standardized structural model is presented in Figure 2, and the direct, indirect, and total associations are summarized in Table 2.

**Table 2:** Standardized direct, indirect, and total associations between curiosity and mathematics achievement.

Effect	Point estimate			95% CI	
	Standardized β	S.E.	*p*-value	Lower (95% CI)	Upper (95% CI)
Effects from interest curiosity to math achievement					
Direct effects	0.113^**^	0.036	0.002	0.043	0.184
Indirect effects	0.210^***^	0.023	< 0.001	0.165	0.255
Interest → CognitiveEng → Math achievement	0.013	0.020	0.525	−0.026	0.052
Interest → BehavioralEng → Math achievement	0.116^***^	0.023	< 0.001	0.072	0.160
Interest → EmotionalEng → Math achievement	0.058^**^	0.021	0.007	0.016	0.100
Interest → SocialEng → Math achievement	0.024	0.012	0.053	0.000	0.049
Total effects	0.324^***^	0.035	< 0.001	0.254	0.393
Effects from deprivation curiosity to math achievement					
Direct effects	0.053	0.033	0.107	−0.011	0.117
Indirect effects	0.155^***^	0.024	< 0.001	0.108	0.202
Deprivation → CognitiveEng → Math achievement	0.010	0.017	0.526	−0.022	0.043
Deprivation → BehavioralEng → Math achievement	0.098^***^	0.021	< 0.001	0.057	0.138
Deprivation → EmotionalEng → Math achievement	0.029^*^	0.012	0.014	0.006	0.053
Deprivation → SocialEng → Math achievement	0.018	0.010	0.064	−0.001	0.036
Total effects	0.208^***^	0.039	< 0.001	0.131	0.285

CI = confidence interval, ^*^p < 0.05; ^**^p < 0.01; ^***^p < 0.001.

### 4.3 Indirect associations

The indirect-effect analyses showed differentiated pathways linking the two curiosity dimensions to mathematics achievement. For interest-type curiosity, the total indirect association with mathematics achievement was statistically significant (β = 0.210, *p* < 0.001). The specific indirect association through behavioral engagement was significant (β = 0.116, *p* < 0.001), as was the indirect association through emotional engagement (β = 0.058, *p* = 0.007). In contrast, the indirect associations through cognitive engagement (β = 0.013, *p* = 0.525) and social engagement (β = 0.024, *p* = 0.053) were not statistically significant. The total association between interest-type curiosity and mathematics achievement was significant (β = 0.324, *p* < 0.001), and the direct association remained significant (β = 0.113, *p* = 0.002).

For deprivation-type curiosity, the total indirect association with mathematics achievement was also statistically significant (β = 0.155, *p* < 0.001). Significant specific indirect associations emerged through behavioral engagement (β = 0.098, *p* < 0.001) and emotional engagement (β = 0.029, *p* = 0.014). The indirect associations through cognitive engagement (β = 0.010, *p* = 0.526) and social engagement (β = 0.018, *p* = 0.064) were not statistically significant. The total association between deprivation-type curiosity and mathematics achievement was significant (β = 0.208, *p* < 0.001), whereas its direct association was not (β = 0.053, *p* = 0.107).

Overall, H1a was supported, whereas H1b was not supported for the direct associations with mathematics achievement. H2a and H2b were supported. H3b, H3c, and H3d were supported, whereas H3a was not supported. H4b and H4c were supported for both curiosity types, whereas H4a and H4d were not supported.

## 5 Discussion

The present study examined how interest-type and deprivation-type curiosity were connected with mathematics achievement through multiple dimensions of academic engagement among Chinese eighth-grade students. By distinguishing two forms of curiosity and simultaneously modeling cognitive, behavioral, emotional, and social engagement, the study moves beyond a general motivation–achievement account and provides a more differentiated view of how curiosity may be translated into mathematics learning outcomes.

Three findings are especially important. First, both interest-type and deprivation-type curiosity were positively related to all four engagement dimensions, but only interest-type curiosity retained a significant direct path to mathematics achievement. Second, behavioral and emotional engagement emerged as the most consistent mediating pathways for both curiosity types, whereas the evidence for social engagement was weaker. Third, and most theoretically important, cognitive engagement was positively predicted by both forms of curiosity but showed neither a significant unique path to achievement nor a significant indirect effect. This pattern suggests that the central question is not simply whether curiosity activates engagement, but whether particular forms of engagement are converted into measurable academic performance.

### 5.1 Differentiated motivational roles of interest-type and deprivation-type curiosity

A central finding of the present study is that interest-type and deprivation-type curiosity were not functionally equivalent. Although both forms of curiosity showed positive total relationships with mathematics achievement, their structural patterns differed. Interest-type curiosity retained a significant direct path to achievement after accounting for the four engagement dimensions and relevant covariates, whereas deprivation-type curiosity did not.

This distinction is consistent with the theoretical differentiation between curiosity driven by the enjoyment of exploration and curiosity driven by the need to reduce an aversive knowledge gap (Litman, 2005, 2008; Litman and Spielberger, 2003). Interest-type curiosity is characterized by positive anticipation and the intrinsic enjoyment of discovering something new. In mathematics learning, such an orientation may be especially consequential because understanding often requires sustained attention to patterns, relationships, and unresolved problems. Students who experience exploration itself as rewarding may continue investing effort even when immediate success is uncertain.

This interpretation is compatible with self-determination theory, which proposes that relatively autonomous forms of motivation are more likely to sustain self-initiated participation and persistence (Ryan and Deci, 2000, 2020). Interest-type curiosity may therefore function as a relatively self-sustaining motivational resource: students explore because the activity of understanding is itself rewarding. The persistence of a direct path to mathematics achievement suggests that this form of curiosity may support performance through processes not fully captured by the four engagement dimensions considered here, such as voluntary exploration, sustained attention to complex problems, or repeated exposure to mathematical ideas. This interpretation is broadly consistent with research linking intellectual curiosity to academic achievement and long-term learning outcomes (von Stumm et al., 2011).

Deprivation-type curiosity displayed a different pattern. Its direct path to mathematics achievement was not significant, whereas its total indirect effect was significant. This finding is consistent with information-gap theory, according to which awareness of missing knowledge generates motivational tension but does not by itself guarantee productive learning (Loewenstein, 1994). A student may strongly want to know why a solution is incorrect or how an unfamiliar problem can be resolved, yet this motivational tension only becomes academically consequential if it is converted into sustained learning activity.

The contrast between the two curiosity types therefore refines a simple “more curiosity is better” account. Interest-type curiosity showed a broader pathway structure, combining a direct path with engagement-related indirect paths. Deprivation-type curiosity appeared more dependent on intermediate learning processes. In theoretical terms, the distinction may lie less in whether both forms motivate students to learn and more in how reliably that motivation is transformed into productive participation.

### 5.2 Engagement as the translational process between curiosity and achievement

The mediation results suggest that curiosity was not translated into mathematics achievement uniformly across all forms of engagement. Rather than four equivalent mediating channels, the pattern was clearly differentiated: behavioral and emotional engagement emerged as the most consistent pathways, social engagement played a weaker role, and cognitive engagement did not mediate either curiosity–achievement relationship.

This pattern supports a process-oriented interpretation of the motivation–engagement–achievement relationship. Curiosity may initiate attention toward unfamiliar information or unresolved problems, but academic consequences appear to depend on whether this motivational orientation becomes sustained participation. Behavioral engagement was the strongest pathway in the model, suggesting that curiosity becomes particularly consequential when students persist, complete tasks, maintain attention, and remain actively involved in mathematics learning. This is consistent with multidimensional engagement theory, which treats behavioral participation as a proximal manifestation of students' investment in learning (Fredricks et al., 2004).

The prominence of behavioral engagement is also plausible given the cumulative structure of mathematics. Mathematical competence develops through repeated encounters with concepts and problems, opportunities to practice, feedback on errors, and sustained participation over time. In such a context, motivational interest alone may be insufficient; students must continue working when problems become difficult. The fact that behavioral engagement mediated both curiosity types suggests a point of convergence between otherwise distinct motivational tendencies. Interest-type curiosity may sustain activity because exploration is enjoyable, whereas deprivation-type curiosity may sustain activity because unresolved uncertainty remains psychologically salient. Despite different motivational origins, both appear capable of being translated into persistent action.

Emotional engagement constituted a second robust pathway. This finding is theoretically important because curiosity itself is closely tied to epistemic emotion. Interest-type curiosity is typically accompanied by enjoyment and exploratory anticipation, whereas deprivation-type curiosity may involve tension generated by incomplete understanding. Research on epistemic emotions suggests that curiosity, confusion, frustration, and enjoyment unfold dynamically during learning rather than functioning as isolated states (Muis et al., 2015; Di Leo et al., 2019). The present findings extend this perspective by suggesting that the academic relevance of curiosity may depend partly on whether students remain emotionally invested in the learning process.

For interest-type curiosity, the emotional pathway is relatively intuitive: enjoyment of exploration may reinforce positive affect and continued participation. For deprivation-type curiosity, the result is more theoretically informative. The discomfort of not knowing may not be beneficial in itself; its academic value may depend on whether students remain engaged with the problem rather than withdrawing. Emotional engagement may therefore represent a regulatory condition under which uncertainty-driven motivation remains productive.

Social engagement was positively associated with mathematics achievement, but its specific indirect associations were not statistically significant. This distinction is theoretically useful. It suggests that socially engaged students may perform better without social engagement necessarily serving as a robust pathway through which curiosity is translated into achievement. The value of social participation may depend more on the quality of interaction than on participation itself. Explaining reasoning, challenging a peer's solution, or jointly constructing an argument is likely to be more academically consequential than interaction that is merely frequent or socially pleasant (Johnson and Johnson, 2009).

Taken together, these results suggest that engagement should not be treated as a homogeneous mediator. Curiosity appears to be most consistently translated into achievement through forms of engagement that sustain actual participation and emotional investment. This shifts the theoretical emphasis away from a generic mediation claim and toward a more differentiated question: which forms of engagement are sufficiently proximal to performance to carry motivational differences into measurable achievement?

### 5.3 Why did cognitive engagement fail to mediate?

The most theoretically challenging finding was the consistent absence of a cognitive engagement pathway. Both interest-type and deprivation-type curiosity positively predicted cognitive engagement, yet cognitive engagement showed neither a significant unique path to mathematics achievement nor a significant indirect effect. This is not a trivial null finding. It creates an important theoretical puzzle because curiosity is often conceptualized as fundamentally cognitive, and cognitive engagement is typically assumed to be one of the primary mechanisms through which motivation supports learning (Fredricks et al., 2004; Zimmerman, 2000).

The first point to clarify is that the null mediation did not arise because curiosity failed to activate cognitive engagement. On the contrary, both curiosity types showed significant positive paths to cognitive engagement. Students who reported higher enjoyment-oriented curiosity and greater sensitivity to knowledge gaps also reported more strategic and meaning-oriented involvement in mathematics learning. The breakdown therefore occurred in the second part of the proposed pathway: self-reported cognitive engagement did not explain unique variation in mathematics achievement once behavioral, emotional, and social engagement were modeled simultaneously.

This distinction is theoretically important. It suggests that curiosity may indeed stimulate cognitive investment, but that such investment does not automatically translate into higher performance. In other words, the present findings challenge a simple assumption that deeper self-reported thinking necessarily constitutes the most effective route from curiosity to achievement.

One explanation concerns the statistical meaning of the cognitive engagement coefficient within a parallel mediation model. The four engagement dimensions were strongly interrelated. Under these conditions, the path from cognitive engagement to achievement represents its unique contribution after variance shared with behavioral, emotional, and social engagement has been partialled out. A non-significant path therefore should not be interpreted as evidence that cognitive engagement is irrelevant. Rather, achievement-relevant variance in cognitive engagement may overlap substantially with other forms of engagement.

This possibility is consistent with multidimensional engagement theory. Cognitive, behavioral, and emotional engagement are conceptually distinguishable, but they are not independent in actual learning situations (Fredricks et al., 2004). A student who actively connects new mathematical ideas with prior knowledge may also persist longer, remain more attentive, and experience greater interest. Once these correlated processes are entered simultaneously, little unique variance may remain for self-reported cognitive engagement to explain. The present result may therefore reflect functional interdependence among engagement dimensions rather than the absence of cognitive involvement.

A second explanation concerns measurement. The study assessed cognitive engagement through self-report. Such measures capture students' perceptions of strategy use, meaning-oriented processing, and cognitive investment, but they do not directly assess whether those strategies are applied accurately, efficiently, or adaptively. This distinction is especially important in mathematics. A student may report trying to connect new information with prior knowledge or thinking deeply about a problem while still relying on ineffective representations, inappropriate procedures, or poorly timed strategies.

Thus, self-reported cognitive engagement may capture perceived effort toward deep learning rather than the quality of actual cognitive processing. This measurement issue offers a plausible explanation for why curiosity predicted cognitive engagement while cognitive engagement did not predict objective examination performance. The former relationship may reflect students' motivational willingness to think deeply; the latter requires that this thinking be effective.

A third explanation concerns the alignment between the mediator and the outcome. Mathematics achievement in the present study was based on a unified end-of-term examination. Although the use of the same test paper across the six schools strengthens comparability, a conventional examination score may be more sensitive to accumulated practice, task completion, procedural fluency, and persistence than to students' self-reported deep-processing tendencies. Cognitive engagement may be more strongly reflected in outcomes requiring transfer, open-ended reasoning, proof, adaptive strategy selection, or novel problem solving.

This interpretation is compatible with research emphasizing that the effectiveness of cognitive and metacognitive strategies depends on task demands and assessment conditions (Zimmerman, 2000). It also provides a more cautious explanation than attributing the null result simply to an “examination-oriented Chinese context.” The present study did not directly measure examination orientation, instructional style, or the cognitive demands of individual test items. Therefore, any contextual explanation should remain tentative.

A fourth possibility is that the relation between cognitive engagement and achievement is temporally delayed. Behavioral engagement can affect performance relatively quickly through increased practice and task completion, whereas the benefits of deep processing and strategic learning may accumulate over longer periods. A cross-sectional design may therefore be less sensitive to cognitive pathways whose effects unfold gradually. Longitudinal research would be needed to determine whether cognitive engagement predicts later achievement change even when its concurrent unique association is weak.

Taken together, these explanations suggest a more precise interpretation of the null finding. The present results do not show that cognitive engagement is unimportant. Rather, they indicate that self-reported cognitive engagement did not explain unique concurrent variance in standardized mathematics achievement once overlapping engagement processes were considered simultaneously. This distinction is theoretically consequential because it separates three processes that are often conflated: being motivated to think deeply, actually engaging in effective cognitive processing, and demonstrating performance on a formal assessment.

The null mediation therefore contributes to the broader model rather than simply representing a failed hypothesis. It reveals a potential discontinuity in the assumed motivation–cognition–achievement sequence. Curiosity appears to stimulate perceived cognitive investment, but this investment may require additional conditions—such as effective strategy use, suitable task demands, or sufficient time—to become visible in achievement outcomes.

### 5.4 Theoretical implications

The present findings contribute to motivation and engagement research in three related ways.

First, they show that curiosity should not be conceptualized as a unitary motivational resource. Interest-type and deprivation-type curiosity both activated broad engagement, yet their routes to mathematics achievement differed. Interest-type curiosity retained a direct path alongside indirect pathways, whereas deprivation-type curiosity depended more heavily on engagement-related processes. This distinction extends two-dimensional accounts of curiosity by showing that different reasons for wanting to know may lead to different achievement structures.

Second, the findings refine the general motivation–engagement–achievement framework. Engagement did not function as a single undifferentiated mediator. Behavioral and emotional engagement emerged as the most stable pathways, social engagement played a weaker role, and cognitive engagement failed to mediate despite being positively predicted by both curiosity types. Thus, the central theoretical contribution is not merely that engagement “mediates” curiosity and achievement, but that the translation of curiosity into achievement is selective across engagement dimensions.

Third, the findings challenge the assumption that cognitively oriented engagement is necessarily the most direct bridge between curiosity and performance. The positive paths from both curiosity types to cognitive engagement indicate that curiosity does stimulate perceived cognitive investment. Yet the absence of a subsequent achievement path suggests that motivational activation and effective performance are distinct stages. This distinction may help reconcile why curiosity can promote students' willingness to think deeply without guaranteeing that such thinking produces immediate achievement gains.

Taken together, the study supports a differentiated motivation–engagement–achievement account in which the academic significance of curiosity depends on both its motivational quality and the engagement process through which it is expressed. Interest-type and deprivation-type curiosity share a broad capacity to activate engagement, but they differ in how strongly they are connected with achievement. Moreover, not all forms of engagement are equally effective in carrying motivational differences into performance. Behavioral and emotional engagement appear to provide the most consistent pathways in the present sample, whereas cognitive engagement may depend more strongly on strategy quality, task characteristics, and temporal development.

Because the study was cross-sectional, these pathways should not be interpreted as established causal mechanisms. Nevertheless, the differentiated pattern of direct and indirect effects provides a theoretically informative basis for future longitudinal and process-oriented research examining how curiosity unfolds into engagement and achievement over time.

### 5.5 Educational implications

The present findings offer several implications for mathematics education, while these implications should be interpreted cautiously given the cross-sectional design of the study. Rather than suggesting that curiosity alone is sufficient to improve achievement, the results indicate that its educational value may depend on how it is expressed through students' engagement with mathematics.

First, mathematics instruction may benefit from creating conditions in which curiosity is translated into sustained behavioral participation. Behavioral engagement emerged as the strongest and most consistent pathway to achievement for both interest-type and deprivation-type curiosity. Accordingly, curiosity-eliciting activities may be most educationally useful when they are accompanied by opportunities for students to persist, revisit unresolved problems, test alternative strategies, and complete extended mathematical tasks. Open-ended questions, problem-posing activities, and tasks that expose meaningful gaps in understanding may stimulate curiosity, but their value is likely to depend on whether classroom structures support continued engagement rather than brief interest alone. Teachers may therefore combine curiosity-eliciting prompts with scaffolds for persistence, such as staged hints, opportunities for revision, and structured follow-up tasks.

Second, the significant role of emotional engagement suggests that mathematics instruction should attend not only to whether students are cognitively challenged but also to how they experience that challenge. Curiosity often arises from uncertainty, novelty, or incomplete understanding, yet these conditions can also generate frustration or withdrawal. Classroom practices that normalize uncertainty, encourage exploratory errors, and provide emotionally supportive feedback may help students remain engaged when solutions are not immediately available. This implication is especially relevant for deprivation-type curiosity, whose potential educational value may depend on whether the tension of “not knowing” is sustained as productive involvement rather than converted into disengagement.

Third, the findings caution against treating all engagement dimensions as interchangeable targets for intervention. Social engagement was positively related to mathematics achievement, but its specific mediating role was comparatively weak in the primary model. This suggests that increasing interaction alone may be insufficient. Collaborative activities are more likely to be educationally meaningful when peer interaction is organized around mathematical explanation, justification, comparison of strategies, and joint problem solving. The pedagogical emphasis should therefore be placed on the epistemic quality of interaction rather than on participation frequency alone.

Finally, the non-significant cognitive mediation pathway has important practical implications. The finding should not be interpreted as evidence that deep thinking or strategic learning is unimportant. Instead, it suggests that encouraging students to report greater cognitive investment may not be sufficient unless they also develop effective and contextually appropriate strategies. Instructional support for cognitive engagement should therefore focus on the quality of strategy use—for example, selecting suitable representations, monitoring solution progress, comparing alternative approaches, and evaluating whether a strategy is effective for a particular task. In addition, cognitively demanding activities may need to be aligned with assessment practices that capture transfer, reasoning, and adaptive problem solving rather than relying solely on conventional performance indicators.

Taken together, the findings suggest that curiosity-oriented mathematics instruction may be most effective when it is embedded within a broader engagement-supportive environment. The practical objective is not simply to make students more curious, but to help them convert curiosity into sustained participation, productive emotional involvement, and effective learning activity. Because the present study is cross-sectional, these implications should be regarded as theoretically informed directions for instructional design rather than as evidence of intervention effects.

### 5.6 Limitations and future directions

Several limitations should be considered when interpreting the findings. First, the cross-sectional design does not permit conclusions about temporal ordering or causal mediation. Although the hypothesized model specified curiosity as antecedent to engagement and mathematics achievement, reciprocal or bidirectional relationships remain possible. For example, higher-achieving students may subsequently experience greater curiosity or engagement. Future research should therefore employ multi-wave longitudinal designs, cross-lagged approaches, or experimental interventions to examine how curiosity and engagement unfold over time.

Second, curiosity and academic engagement were assessed using self-report measures. Although all items were contextualized specifically to mathematics learning and mathematics achievement was obtained independently from official examination records, shared method variance may still have inflated some associations among the self-reported constructs. Future studies should integrate multiple sources of evidence, such as classroom observation, teacher ratings, learning analytics, experience-sampling methods, or task-based indicators of engagement. Although the translated items were reviewed by the research team for semantic equivalence and age appropriateness, the adaptation process did not include an independent back-translation or formal cognitive interviewing with students. Further cross-cultural validation of the adapted measures is therefore warranted.

Third, the present study included home book resources and maximum parental educational attainment as available proxy indicators of socioeconomic and educational background. These covariates strengthen the analysis by accounting for important family-background differences, but they do not constitute a comprehensive measure of socioeconomic status such as ESCS. Future research should incorporate broader indicators including household income, parental occupation, educational resources, and other contextual characteristics.

Fourth, the sample consisted of eighth-grade students from six junior secondary schools in Qinghai Province, China. Although the participating schools provided variation within a western Chinese educational context, the findings may not generalize directly to students from other regions, age groups, school systems, or cultural settings. Replication across more diverse populations is therefore needed to determine whether the differentiated pathways identified here remain stable across educational contexts.

Finally, the non-significant role of cognitive engagement should be interpreted in light of measurement and outcome alignment. Self-reported cognitive engagement may not capture the quality, adaptiveness, or moment-to-moment enactment of cognitive strategies during mathematical problem solving. In addition, the unified end-of-term examination may not fully represent outcomes such as transfer, adaptive reasoning, or complex problem solving, where cognitive engagement may be more consequential. Future research should combine self-report measures with process-based assessments and more cognitively differentiated achievement outcomes.

In addition, the analysis did not explicitly account for the nesting of students within classes and schools. Because the study included only six schools, school-level effects could not be estimated reliably. Future research should recruit a larger number of schools and apply multilevel or cluster-robust approaches to distinguish student-, classroom-, and school-level variation.

Overall, future studies should move beyond concurrent mediation models and examine when, for whom, and under what instructional conditions different forms of curiosity are translated into particular engagement processes and subsequent mathematics outcomes.

## 6 Conclusions

This study examined the mediating pathways linking interest-type and deprivation-type curiosity to mathematics achievement among Chinese eighth-grade students. By distinguishing two forms of curiosity and four dimensions of academic engagement, the study provides a more differentiated account of the motivation–engagement–achievement relationship in mathematics learning.

The findings showed that both forms of curiosity were positively related to cognitive, behavioral, emotional, and social engagement. However, their pathways to achievement differed. Interest-type curiosity retained a significant direct path to mathematics achievement, whereas deprivation-type curiosity did not. Behavioral and emotional engagement emerged as the most consistent mediating pathways for both curiosity types. In contrast, cognitive engagement did not significantly mediate either relationship, and the specific indirect associations through social engagement were not statistically significant.

These findings suggest that the academic significance of curiosity depends not only on whether students are curious, but also on the form of curiosity and the type of engagement through which it is expressed. In particular, curiosity appears to be more consistently connected with achievement when it is translated into sustained behavioral participation and emotional involvement. The absence of a significant cognitive mediation pathway further indicates that motivational willingness to think deeply should not be equated automatically with effective cognitive processing or measurable performance.

The study therefore contributes to a more selective and process-oriented understanding of how curiosity is connected with mathematics achievement. Rather than supporting a single general pathway from motivation to performance, the results point to differentiated routes across curiosity types and engagement dimensions. Because the study was cross-sectional, these pathways should be interpreted as model-based indirect effects rather than established causal mechanisms. Future longitudinal and process-oriented research is needed to clarify how these relationships unfold over time and under different instructional conditions.

## Data Availability

The data that support the findings of this study are available from the corresponding author upon reasonable request, subject to applicable ethical and privacy restrictions.
